# Mortality of a postpartum woman presented with massive vulvar edema in association with Covid-19: a case report with clinical and radiological findings

**DOI:** 10.1186/s12879-021-06175-8

**Published:** 2021-07-13

**Authors:** Somayeh Alirezaei, Atiye Vatanchi, Leila Pourali, Behzad Aminzadeh, Robab Latifnejad Roudsari

**Affiliations:** 1 Student Research Committee, School of Nursing and Midwifery, Mashhad University of Medical Sciences, Mashhad, Iran; 2 Department of Obstetrics and Gynecology, Faculty of Medicine, Mashhad University of Medical Sciences, Mashhad, Iran; 3 Department of Radiology, Faculty of Medicine, Mashhad University of Medical Sciences, Mashhad, Iran; 4 Nursing and Midwifery Care Research Center, Mashhad University of Medical Sciences, Mashhad, Iran; 5 Department of Midwifery, School of Nursing and Midwifery, Mashhad University of Medical Sciences, Mashhad, Iran

**Keywords:** Vulvar diseases, Postpartum women, SARS-CoV-2, Case report

## Abstract

**Background:**

In this case report, we presented a rare case of maternal death with massive vulvar edema and Covid-19 diagnosis.

**Case presentation:**

The case was a 20-year-old woman who was referred to with pain and massive vulvar edema by passing 7 days from her labor. The laboratory tests showed leukocytosis, lymphopenia, and elevated C-reactive protein levels. The high-resolution computed tomography was in favor of Covid-19 changes. Finally, she died because of respiratory distress, ON the 8th day postpartum.

**Conclusion:**

Given the increasing prevalence of Covid-19, it is important and vital to be aware of its potential complications and then to try prevent and manage them, especially during high-risk periods such as pregnancy and postpartum.

## Background

WHO announced the coronavirus disease 2019 as a pandemic [1]. Accordingly, the most common symptoms of this disease are fever and cough; however [2], a wide range of symptoms have been reported so far. Sepsis, respiratory failure, ARDS, acute heart failure , and acute kidney injury are accompanied by this infection [3]. Pregnant women are at high risk of increasing a viral infection such asSARS-CoV, MERS-CoV, and influenza [4] Common symptoms at the start of respiratory infection in pregnant women include fever and cough, malaise, myalgia sore throat, dyspnea, and diarrhea. Lymphopenia is indicated in laboratory checks [5]. Based on initial reports largely from China, severe acute respiratory syndrome coronavirus 2 (SARS-CoV-2) does not appear to follow these historical patterns of worsened disease risk in pregnancy [6] During three of the influenza pandemics of the last years (1918, 1957–1958, 2009), pregnant women in their second or third trimester were significantly more likely to be hospitalized or expire compared with the overall people [7]. in a detailed case series of 9 gravida from Iran, Among 9 pregnant women with severe COVID-19 disease, at the time of reporting, 7 of 9 died, 1 of 9 remains critically ill and ventilator dependent, and 1 of 9 recovered after prolonged hospitalization [8]. Various studies have shown the effects of new -onset infections on maternal issues; including maternal mortality, spontaneous abortion, and preterm delivery compared to non-pregnant women [5]. There are inadequate data on the effects of Covid-19 on maternal complications and the clinical characteristics and vertical transmission potential of COVID–19 pneumonia in pregnant women are unknown [9]. So, it seems necessary to report various presentations of this novel virus on maternal outcomes. Therefore, in this case report, a rare case of maternal death with massive vulvar edema and Covid-19 diagnosis in early postpartum was presented.

## Case presentation

**Fig. 1:**
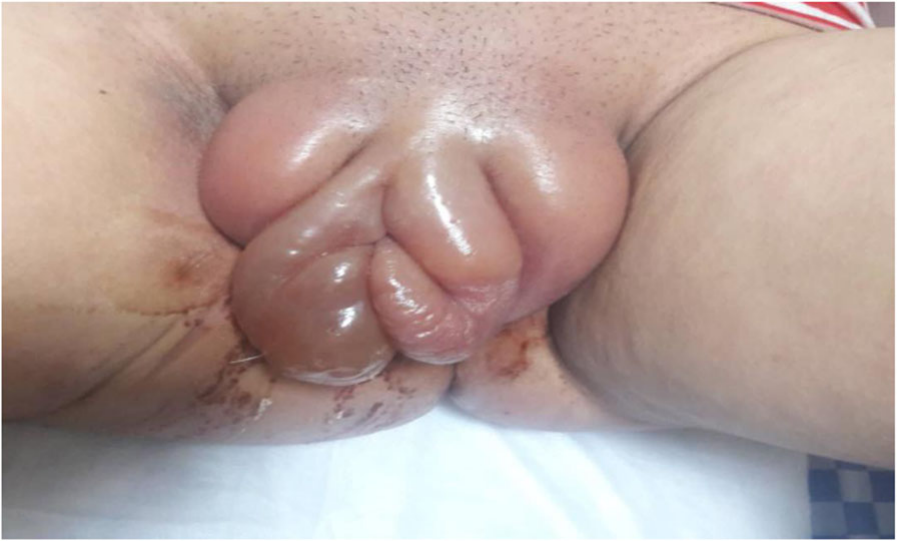
Massive vulvar edema in patient with severe

A 20-year-old previously healthy gravida (G1P1) was referred with sever vulvar pain and edema (Fig. 1) on the 7th day after her labor. The past medical history of the patient showed no presence of anemia (Hb: 13.3, HCT: 39.2, BGRH: B+) high blood pressure, malnutrition, hemoglobinopathies, lower limb edema , or any complications such as symptoms of covid-19. She gave a normal vaginal delivery with restrictive episiotomy incision at 40 weeks of gestation 7 days before the admission without any complication. After that, she experienced mild vulvar edema by passing 12 h from labor. Thereafter, she was hospitalized for 2 days. She was then discharged with the administration of some medications including Acetaminophen, Cefixime, Metronidazole, and Enoxaparin, and by considering that no basic cause was found for her vulvar edema.

Four days after discharge, the patient came back to the hospital with the chief complain of sever vulvar edema (Fig. 1). Subsequently, the vital signs showed hypothermia and bradycardia (T: 35.5, PR: 56. BP: 120/70, RR: 24 & SPO2:97%). The patient also had an ill and dehydrated appearance, anorexia, and oliguria accompanied by mild nausea in the last few days. The patient reported no symptoms of respiratory disease. She was then hospitalized with the initial diagnosis of necrotizing fasciitis or cellulitis.

**Fig. 2:**
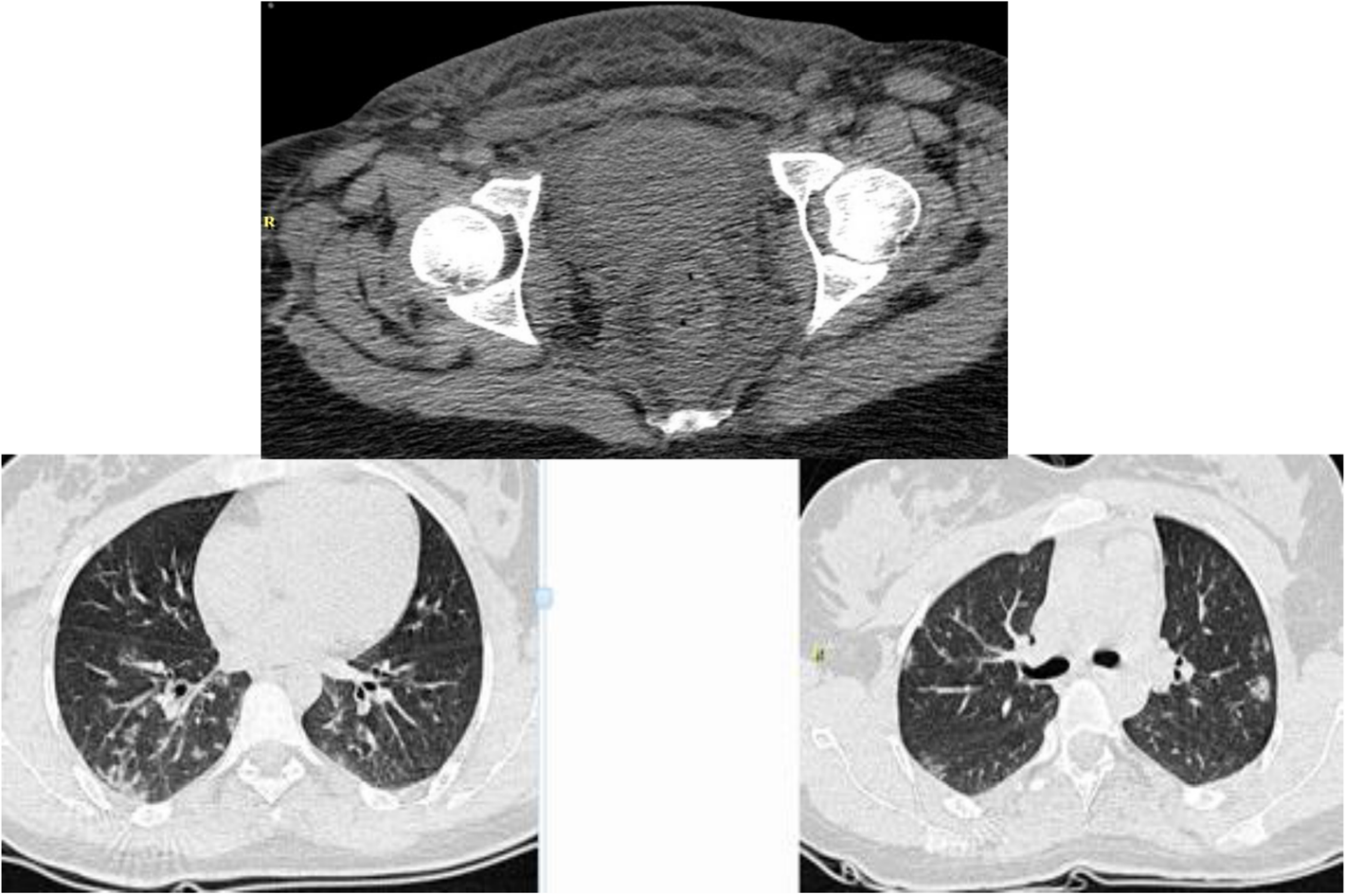
Multiple bilateral, multi-lobar, peripheral and round ground glass opacities

The clinical examination indicated severe and uncommon edema in the hypogastric region spreading to the perineal and gluteal regions (Based on the image shown in Fig. 1) with tenderness and exudative discharge; however, the episiotomy incision had a normal appearance. The spiral Lung HRCT , as well as Spiral CT of the abdomen and pelvis , werewas requested to examine the subcutaneous emphysema and the necrotizing fasciitis. In CT of the abdomen and pelvis, there were edema and stranding in the subcutaneous soft tissue of the hypogastric region, preferably in the pelvis, free fluid in the abdomen, and stranding in the pelvis. In HRCT multiple bilateral, multi -lobar, peripheral, and round ground -glass opacities were seen. Altogether, these findings were highly suggestive for Covid-19 pneumonia (typical appearance) (Fig. 2).

The antibiotic regimen of Meropenem and Vancomycin, azithromycin and Caletra (Lopinavir & Ritonavir), and Hydroxychloroquine were added to the patient’s medication regimen due to the high prevalence of coronavirus in society and hospital.

On  the same day, the patient was transferred to the operating room with the possibility of necrotizing fasciitis. Thereafter, to overcome the oliguria, the CVL was installed for the patient in the operating room. Edematous and thick perineal skin debridement was performed and a clear, non-purulent transudate was removed from the cutaneous and subcutaneous tissues. Moreover, a sample was sent for culture and antibiogram and based on the obtained results, the possibility of necrotizing fasciitis was rejected by the surgeon, due to tachycardia and rhythm 130, the echocardiography was ordered in bed, which consequently resulted in the systolic dysfunction (EF = 40%), global hypokinesis, and mild hypertrophy of the left ventricle.

Due to positive RTPCR testing for covid-19, leukocytosis, the initial NLR > 3.5 and CRP = 78 mg/L, and the Lung HRCT report, the patient was quickly sent to the isolated ICU with the suspected pneumonia of Covid-19 and sepsis. Despite sustainable vital signs, diuresis, and normal Oxygen saturation, the patient had mild respiratory distress. Repeated tests indicated hypoalbuminemia, higher INR, thrombocytopenia, leukocytosis, severe lymphopenia, greater lactate dehydrogenase, hyperbilirubinemia, metabolic acidosis, and progressive hepatocellular damage. Afterward, the hematologists checked the peripheral blood smear, excluded leukemia, and finally confirmed the leukemoid reaction. Notably, the patient was severely dehydrated. In addition, the results of repeated laboratory tests showed sever leukocytosis and hemoconcentration. Accordingly, C Albumin was prescribed to correct the condition.

 On the morning of the next day, the patient’s respiratory problems increased and the oxygen saturation level decreased from 97 to 87%. Four hours later, the patient had apnea, so she was immediately intubated and connected to a ventilator. Mydriasis was doubled at the same time, and Epinephrine drip continued for her. An hour later, she underwent CPR due to hemodynamic disorder, respiratory arrest, and bradycardia. Finally, she was expired because of respiratory distress after 33 h of hospitalization.

## Discussion and conclusions

Since 2000, two other outbreaks of coronavirus, except Covid-19, including SARS-CoV [10] and MERS-CoV [11] have occurred. Due to the emergence of the Covid-19 virus, there are few studies conducted on its maternal side effects. However, evaluations performed on the effects of the prevalence of previous coronaviruses in the antepartum period indicated a high risk of death in this group. Notably, it was reported that the mortality rates for SARS and MERS were 25 and 40%, respectively [10, 11].

Despite the fact that edema is common among those women who have given birth, severe edema is limited to labia in few women. However, vulvar edema can occur in one or two-sided shapes. In the present paper, we reported a case of Covid-19 with severe and bilaterally vulvar edema after delivery. In this regard, the causes of severe vulvar edema included inflammatory trauma disorders and infection [12].

The radiography findings indicated the edema, stranding, and inflammation in the subcutaneous soft tissue of the hypogastric region, preferably in the pelvis and ground glass patches of lower lobes of lungs, which were diagnosed at the early acute stages of the disease [13]. It is noteworthy that during the pregnancy, a significant increase in blood volume, blood pressure, and vascular permeability along with the compression of the IVC following the uterine growth can cause edema Besides respiratory effects, cardiovascular effects of the new coronavirus disease also are important threats. Despite the exact mechanism and pathophysiology of infection caused by Covid-19, there is no complete understanding of it yet [3], However, the focal effects on pulmonary edema, cerebral edema, liver damage, and destruction [14] all are significant risks of Covid-19. In addition, it was indicated that the lower renal function can also affect the serum protein concentration and salt retention [15]. Other related mechanisms, including venous congestion [16], similar to other vascular manifestations of Covid-19, also increase the capillary pressure and cause the incomplete vascular reflex. Many of these mechanisms caused by the coronavirus, may consequently lead intravascular fluid to leak into the interstitial space and cause systemic or localized edema [17].

Also, severe vulvar edema might be a sign of severe coronavirus disease. Reports of postpartum vulvar edema syndrome indicate up to 80% of maternal deaths [18]. Conservative measures are the most commonly reported treatment of vulvar edema, which is usually associated with spontaneous recovery after delivery [19]. Conservative treatment is not recommended when the life of the mother or fetus is at risk, such as patients with severe coronavirus disease. In addition, conservative edema management cannot prevent necrosis and tissue destruction، as it progresses. Few physicians have reported using surgical procedures to manage severe vulvar edema [20, 21]. As aforementioned, the incisions in the affected region in our patient could reduce the fluid pressure and facilitate the blood flow in the edematous regions. Lyndsey et al. (2014) recommended that surgery should be considered if the conservative treatment is not successful or the prompt action is needed to prevent adverse maternal outcomes [17]. Prolonged edema can result from microvasculature destruction arising from Covid-19. In a study by Yao et al. (2020), edema was reported among clinical manifestations in some patients with Covid-19 [22]. In the present case, 7 days were spent after the onset of edema in the patient, and she was in a critical condition when she was admitted, and required care and treatment in ICU.

In a study, the average time of hospitalization in the ICU until the mechanical ventilation was mentioned to be 10.5 days [23]; however, the rapid progress of consolidative opacities in our patient only lasted 30 h. Based on statistics, about 5% of patients with SARS-COV-2 have multi-organ dysfunction that results in a mortality rate of 1.4% [2]. Zhu et al. (2020) considered organ failure as the cause of a direct virus attack [24]. However, the question of how SARS-COV-2 can be spread to extra-pulmonary organs still is a secret [25]. It has been reported that vascular endothelial cells are the organs attacked by SARS-COV-2 leading to abnormal coagulation and sepsis by the ACE2 level increasing [26]. Furthermore, the patients in a severe condition of Covid-19 had some critical shock manifestations such as chills, coldness of the peripheral organs, and a weak pulse without any obvious drop in blood pressure. Notably, many patients had metabolic acidosis as a sign of circulatory dysfunction [25]. In addition, hepatic impairment was seen in some of them [27]. Similarly, the case presented in this report had also diagnostic criteria for sepsis and septic shock, which have been defined by the Third International Consensus Definitions for Sepsis [28], however, SARS-COV-2 was the only reason for the emergence of such criteria. Therefore, the viral sepsis can more accurately describe the severe clinical manifestations of a presented case of Covid-19.

To the best of our knowledge, no case of post-partum mortality due to Covid-19 has been reported so far. Given the increasing prevalence of Covid-19 [28], consequently, its possible adverse effects also increase. As mentioned earlier, severe vulvar edema, infection, organ dysfunction, and sepsis could be associated with Covid-19. In such cases, it is important and vital to be aware of the complications and also to try to prevent and manage them timely and appropriately, especially during high-risk periods such as pregnancy, childbirth, and postpartum.

## Data Availability

Not applicable.

## Abbreviations

Covid-19: Coronavirus Disease 2019
WHO: World Health Organization
ARDS: Acute Respiratory Distress Syndrome
G, P: Gravidity, Para
T: Temperature
PR: Pulse Rate
BP: Blood Pressure
RR: Respiratory Rate
SPO2: Oxygen Saturation
HRCT: High-Resolution Computed Tomography
CT: Computed Tomography
CVL: Central Venous Line
NRL: Neutrophil to lymphocyte ratio
ICU: Intensive Care Unit
INR: International Normalized Ratio
EF: Ejection Fraction
CPR: Cardiopulmonary Resuscitation
SARS-CoV2: Severe Acute Respiratory Syndrome Coronavirus
MERS-CoV2: Middle East Respiratory Syndrome Coronavirus
IVC: Inferior Vena Cava
ACE2: Angiotensin-Converting Enzyme 2
HCT: Hematocrit
CRP: C-Reactive Protein
WBC: White Blood Cells
RBC: Red Blood Cells
AST: Aspartate Aminotransferase
ALT: Alanine Transaminase
ALP: Alkaline Phosphatase
LDH: lactate Dehydrogenase
PT: Prothrombin Time
PTT: Partial Thromboplastin Time
PH: Power Hydrogen
PO2: Partial Pressure of Oxygen
PCO2: Partial Pressure of Carbon Dioxide
